# Metabolomic and microbial diversity perspectives on *Aspergillus oryzae* culture-induced modifications in ovine feed utilization and rumen ecosystem

**DOI:** 10.3389/fvets.2025.1658361

**Published:** 2025-11-14

**Authors:** Jinglong Xie, Caidie Wang, Yanhui Zhao, Xiaoming Li, Guijun Ma, Kailun Yang

**Affiliations:** 1 Xinjiang Key Laboratory of Herbivore Nutrition for Meat and Milk, College of Animal Science, Xinjiang Agricultural University, Urumqi, China; 2 Xinjiang Tiankang Feed Co., Ltd., Urumqi, China

**Keywords:** *Aspergillus oryzae* culture, sheep, fiber degradation, rumen metabolism, microbial diversity

## Abstract

**Introduction:**

Aspergillus oryzae culture (AOC) is widely used as a feed additive to enhance ruminant productivity and rumen function. However, the underlying mechanisms at the microbiome-metabolome interface remain poorly understood. This study aimed to elucidate how dietary AOC supplementation influences sheep production performance, rumen fermentation, microbial communities, and metabolomic profiles.

**Methods:**

Twelve rumen-fistulated sheep were randomly assigned to a control group (basal diet) and a trial group (basal diet + 1% AOC). The experiment lasted 30 days, during which production performance, nutrient digestibility, ruminal pH, volatile fatty acids (VFA), ammonia nitrogen, microbial diversity (16S rDNA sequencing), and metabolomic profiles (LC-MS) were systematically assessed.

**Results:**

AOC supplementation significantly increased average daily gain (ADG) and neutral detergent fiber (NDF) digestibility by 7.00% (*p* < 0.05), and improved nitrogen retention. Total VFA and acetate concentrations were elevated, with a stable ruminal pH. Microbiome analysis revealed an increased relative abundance of Succiniclasticum and beneficial fiber-degrading taxa. Metabolomic profiling identified upregulation of antioxidant metabolites (e.g., ginsenoside Rg3, lipoamide) and activation of key pathways such as phenylalanine metabolism and the TCA cycle, alongside downregulation of inflammatory markers.

**Discussion:**

AOC enhances sheep productivity and rumen health by modulating fibrolytic microbiota, promoting VFA synthesis, and activating antioxidant and energy metabolism pathways. These findings provide a theoretical basis for the use of AOC as a sustainable feed additive to improve ruminant production efficiency and welfare.

## 1 Introduction

*Aspergillus oryzae* (*A. oryzae*) is one of the most extensively studied edible fila-mentous fungi, and its commercial applications are widely utilized in the production of soy sauce, sake, and miso (1). *A. oryzae* is also commonly added to animal feed as a direct-fed microbial (DFM), and its fermentation extract Amaferm® has been widely adopted in agricultural applications (2, 3). Research has demonstrated that *A. oryzae* possesses a robust enzymatic system capable of secreting diverse enzymes, such as α-amylase, β-amylase, protease, and cellulase, which enable the breakdown and utilization of various substrates (4). Studies have shown that supplementing ruminant feed with *A. oryzae* enhances feed intake (5), improves fiber and dry matter digestibility (6), and increases the population of fibrolytic bacteria in the rumen. These effects collectively improve rumen digestive function, elevate the concentration of volatile fatty acids (VFAs) as a metabolizable energy source, and enhance milk production efficiency (7). You et al. found that dietary supplementation with 50 g/ (head·d) of *A. oryzae* culture enhanced the apparent digestibility of DM, NDF, and ADF in beef cattle (8). Schingoethe further revealed that adding *Aspergillus oryzae culture* (AOC) not only increases total dry matter intake but also elevates the concentrations of total volatile fatty acids (TVFA) and propionate, while reducing the acetate-to-propionate ratio (9). The mechanisms underlying these benefits are multifaceted, involving not only the exogenous enzymes provided by AOC but also its prebiotic components (e.g., oligosaccharides) and bioactive metabolites that stimulate the host’s endogenous enzyme secretion and gut barrier function (49). Furthermore, the antioxidant properties of AOC’s metabolites, such as kojic acid and various polyphenols, contribute to mitigating oxidative stress and supporting overall animal health and immune status (10, 11). The cumulative evidence underscores AOC’s potential as a sustainable strategy to enhance animal production by synergistically improving digestive efficiency and the microbial ecosystem.

Current research on AOC has primarily focused on their effects on production performance and rumen microbiota, while mechanistic insights at the metabolome-microbiome interface remain largely unexplored. To address this gap, the present study utilizes fistulated sheep as experimental animals, supplementing their diet with AOC. The purpose of this study was to elucidate the mechanism of AOC in sheep by systematically analyzing sheep production parameters, rumen fermentation characteristics, rumen microbial composition and rumen fluid metabolome.

## 2 Materials and methods

### 2.1 Ethical considerations

The experimental procedures, management, and animal husbandry followed the regulations and instructions of the Animal Welfare and Ethics Committee of Xinjiang Agricultural University, Urumqi, Xinjiang, China (Approval number 2020022).

### 2.2 Animals and experimental design

The experiment selected 12 sheep with similar body conditions (39.07 ± 1.78 kg), uniform gender (male), and approximately 8 months of age as experimental animals. Permanent ruminal fistulas were surgically installed in the sheep. The 12 fistulated sheep were randomly divided into two groups: the control group and the trial group, with 6 sheep in each group. The control group was fed a basal diet, while the trial group received a diet supplemented with 1% AOC (provided by Xinjiang Tiankang Feed Co., Ltd., China, AOC contained 1 × 10^9^ CFU/g of *A. oryzae* spores, the enzymatic activity of neutral protease is 3,245.3 IU/g, GB/T23527-2009). After a 7-day adaptation period, the 30-day formal trial commenced. A digestion and metabolism trial involving total fecal and urine collection was conducted from days 25 to 29 of the experiment. AOC material was supplemented as an additional ingredient at 1% of the basal diet and fed to fistulated sheep. During the trial, the management conditions were consistent between the two groups of fistulated sheep. The initial daily feeding amount was set at 1.2 kg per sheep (resulting in a daily intake of *A. oryzae* spores at 1.2 × 10^6^ CFU/g per sheep). Feeding was conducted twice daily at 09:00 and 18:00, with 0.6 kg provided per sheep at each feeding. The concentrate and corn straw were mixed prior to feeding. The feeding amount was adjusted based on leftover feed: if leftovers exceeded 10% of the offered feed or there were no leftovers for two consecutive days, the amount was modified on the third day. Throughout the experiment, the sheep had free access to water.

### 2.3 Data and sample collection and analysis

#### 2.3.1 Production performance

Daily feed offered was recorded for each sheep, and residual feed from the pre-vious day was collected and weighed before the morning feeding. Body weights were measured on days 0 and 30 of the formal trial period prior to morning feeding.

Calculation Formulas: Average Daily Gain (ADG) = (Final Body Weight – Initial Body Weight)/Trial Duration (days); Dry Matter Intake (DMI) = (Feed Offered – Residual Feed) × Dietary Dry Matter Content (%); Feed-to-Gain Ratio (F:G) = DMI/ADG.

#### 2.3.2 Collection and analysis of feed, fecal, and urine samples

The digestion and metabolism trial was conducted from days 25 to 29 of the experiment. Approximately 200 g of forage samples were collected using the five-point sampling method. During the trial period, residual feed from each experimental sheep was collected daily, weighed, air-dried, and then ground through a 40-mesh sieve. The total fecal collection method was employed by fitting custom-made fecal collection bags to the tail of each sheep. Fecal samples were collected every 4 h from all sheep, with fresh weight recorded continuously for 3 days. The daily collected samples were thoroughly mixed, and 10% of the total was preserved. For total urine collection, custom-made urine collection devices were attached to the urination site of the sheep prior to the digestion trial. Urine was collected every 4 h from all animals. The urine samples from each sheep during the trial period were homogenized, with 10% of the total volume preserved using 10% concentrated sulfuric acid for nitrogen fixation.

The moisture and crude protein contents in the feed and feces were determined according to the Chinese National Standards GB/T 6435-2014 and GB/T 6432-2018, respectively. The neutral detergent fiber and acid detergent fiber were analyzed using a fully automated fiber analyzer (ANKOM A2000i). Specific results are presented in Appendix 1.

#### 2.3.3 Rumen fluid sample collection and analysis

Rumen fluid samples were collected via ruminal fistula at 0 h (pre-feeding), 2 h, 4 h, 6 h, 8 h, and 10 h post-morning feeding on days 0, 10, 20, and 30 of the trial. The samples were immediately filtered through four layers of gauze and analyzed using a pH meter. Additional rumen fluid samples (~200 mL) were collected on day 0 (pre-feeding) and day 30 (at 0 h, 2 h, 4 h, 6 h, 8 h, and 10 h post-feeding), filtered similarly, and processed for further analysis. Volatile fatty acids (VFA) were quantified using a Shimadzu GC-2010 gas chromatograph equipped with a Stabilwax column (30 m × 0.25 mm × 0.25 μm), with 4-methylvaleric acid as the internal standard. Ammonia nitrogen (NH_3_-N) concentrations in rumen fluid collected on days 0 and 30 were determined according to the method described in reference (12).

#### 2.3.4 Rumen microbial diversity

On day 30 of the experiment, rumen fluid was collected via ruminal fistula, snap-frozen in liquid nitrogen, and subsequently subjected to 16S rDNA high-throughput sequencing. The specific methods are described in Appendix 2.

#### 2.3.5 Rumen fluid metabolomics profiling and analysis

On day 30 of the experiment, rumen fluid was collected via ruminal fistula, snap-frozen in liquid nitrogen, and subsequently subjected to metabolomic analysis. For methodological details, please refer to Appendix 3.

### 2.4 Statistical analysis

GraphPad 9.5.1 and Origin 2024 was used for figures. Statistical analyses were performed using SPSS 26 software. For all parameters, data between groups were compared using independent samples t-tests with *p* < 0.05 as the significance level.

Metabolites were annotated using the KEGG, HMDB, and LIPIDMaps databases. Differential metabolites were screened with the criteria of VIP > 1, *p* < 0.05, and fold change (FC) ≥ 2 or FC ≤ 0.5. PCA and PLS-DA were performed using metaX. Volcano plots were generated with ggplot2, and clustering heatmaps were plotted using the Pheatmap package with z-score normalized data. Correlation analysis between metabolites was conducted using the cor() function (Pearson method), with significance calculated by cor.mtest() and results visualized via the corrplot package. Pearson correlation analysis was performed between differentially abundant bacterial genera (identified from 16S rDNA sequencing) and differential metabolites (identified from metabolomics). Results were visualized in heatmaps. The correlation coefficient *r* = cov(X,Y)/(σX*σY), where |*r*| values closer to 1 indicate stronger correlations, with *r* > 0 representing positive correlations and *r* < 0 indicating negative correlations (Table 1).

**Table 1:** The composition and nutritional level of the diet.

Items	Content (%)	Nutritional level	Content (%)
Corn	29.58	DM (%)	89.12
Soybean meal	2.27	CP (%)	13.89
Rice bran	5.54	Ash (%)	7.43
Corn germ meal	17.65	EE (%)	2.35
Spray-dried corn gluten feed	30.91	Ca (%)	0.99
Limestone	2.20	P (%)	0.56
Sodium chloride	0.5	NDF (%)	30.78
Sodium bicarbonate	0.45	ADF (%)	16.41
Ammonium chloride	0.45		
Corn stover	10.00		
Premix	0.45		
Total	100.00		

The premix provided the following for per kg concentrate: VA 40,000 IU, VD3 5,000 IU, VE 200 IU, Cu (as copper sulfate) 20.00 mg, Fe (as ferrous sulfate) 150.00 mg, Mn (as manganese sulfate) 120.00 mg, Zn (as zinc sulfate) 150.00 mg. Nutrient levels were determined values.

## 3 Results

### 3.1 Production performance and diet digestibility in sheep

The results regarding the effects of dietary supplementation with AOC on the production performance of fistulated sheep are presented in Table 2. As shown in the table, the average daily gain (ADG) of the trial group was significantly higher than that of the control group (*p* < 0.05). The feed intake showed an increasing trend and the feed conversion ratio (FCR) a decreasing trend in the trial group, though these differences were not statistically significant (*p* > 0.05). In terms of apparent digestibility, AOC supplementation significantly improved the digestibility of neutral detergent fiber (NDF) by 7.00% (*p* < 0.05), but had no significant effects on the digestibility of dry matter (DM), crude protein (CP), or acid detergent fiber (ADF) (*p* > 0.05). There was no significant difference in nitrogen intake between the two groups (*p* > 0.05), however, nitrogen retention was significantly higher in the trial group compared to the control group (*p* < 0.05).

**Table 2:** Effects of dietary supplementation with AOC on production performance and diet digestibility in sheep.

Items	Control group	Trial group	*p*-value
IBW/kg	39.22 ± 1.39	38.92 ± 2.27	0.81
FBE/kg	43.99 ± 1.32	44.36 ± 2.14	0.73
ADG/g	128.95 ± 10.58^b^	147.1 ± 13.52^a^	0.046
DMI/kg	0.80 ± 0.06	0.89 ± 0.05	0.33
F/G	6.26 ± 0.92	6.07 ± 0.77	0.73
DM/%	69.83 ± 6.85	71.41 ± 5.36	0.68
CP/%	67.11 ± 5.50	70.05 ± 5.48	0.42
NDF/%	54.42 ± 2.32^b^	58.23 ± 2.61^a^	0.04
ADF/%	47.34 ± 4.40	50.51 ± 1.11	0.21
N uptake (g/d)	14.91 ± 1.8	15.15 ± 2.52	0.87
Deposited N (g/d)	7.89 ± 1.03	8.64 ± 0.71	0.23
N deposition rete/%	50.41 ± 6.31^b^	58.20 ± 3.55^a^	0.046

SEM, standard error of means. a, b means within a row with different superscripts differ significantly (*p* < 0.05).

### 3.2 Rumen fermentation

#### 3.2.1 Rumen pH

**Figure 1:**
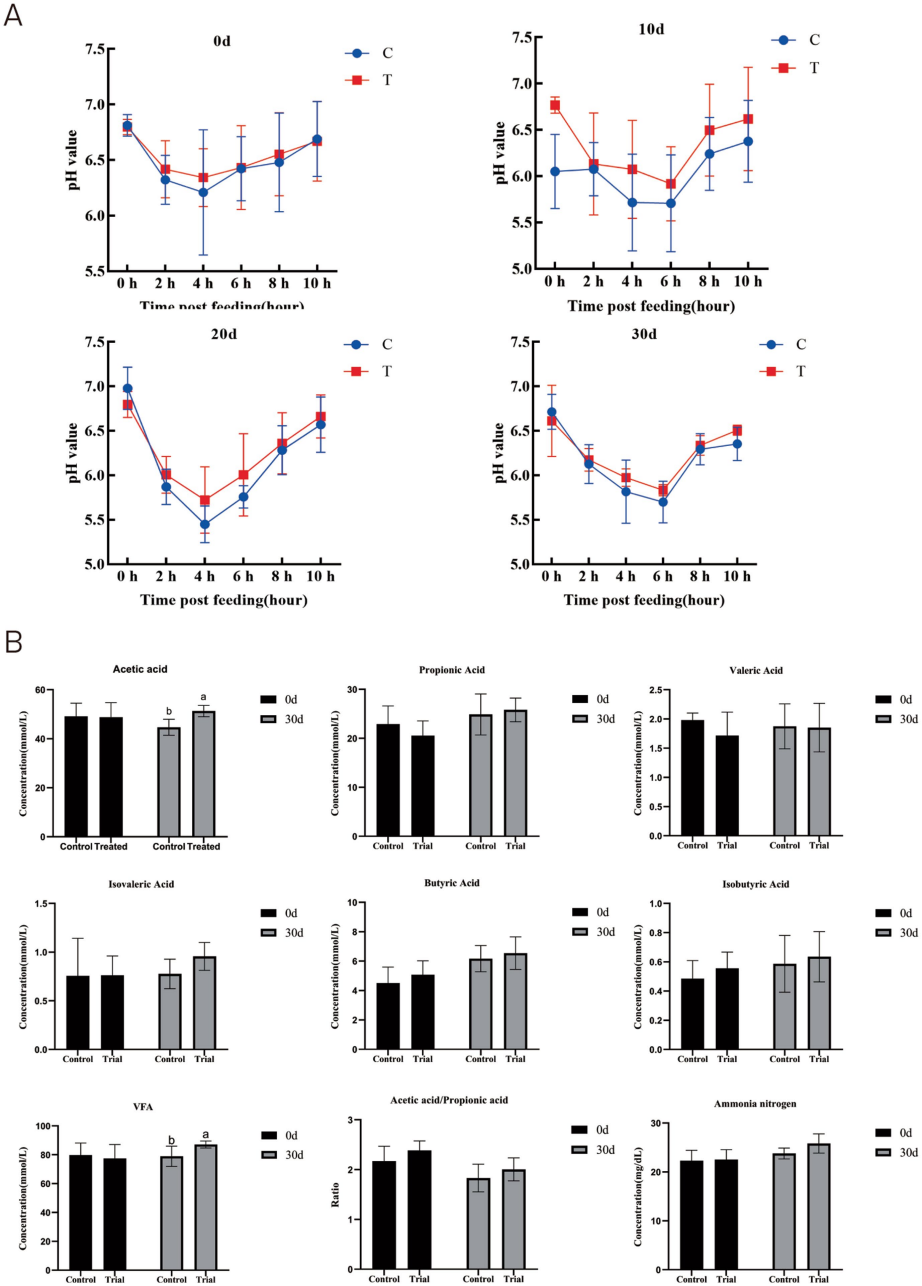
Dynamic changes in ruminal pH at different time points **(A)** and effects on ruminal VFA and ammonia nitrogen concentrations **(B)**. a, b means within a figure with different superscripts differ significantly (*p* < 0.05).

Following AOC supplementation, the ruminal pH dynamics in sheep showed an initial decrease followed by a gradual increase (Figure 1A). The ruminal pH values in the trial group were higher than those in the control group at 2 h, 4 h, and 6 h post-feeding, indicating a trend toward increased ruminal pH with AOC supplementation.

#### 3.2.2 Rumen VFA and ammonia nitrogen

On day 0, there were no significant differences in volatile fatty acid (VFA) or NH_3_–N concentrations between the two groups (*p* > 0.05, Figure 1B). By day 30, the acetate and total VFA concentrations in the trial group were significantly higher than those in the control group (*p* < 0.05), while the NH_3_–N level was 8.59% higher than that of the control, though this difference was not statistically significant (*p* > 0.05).

**Figure 2:**
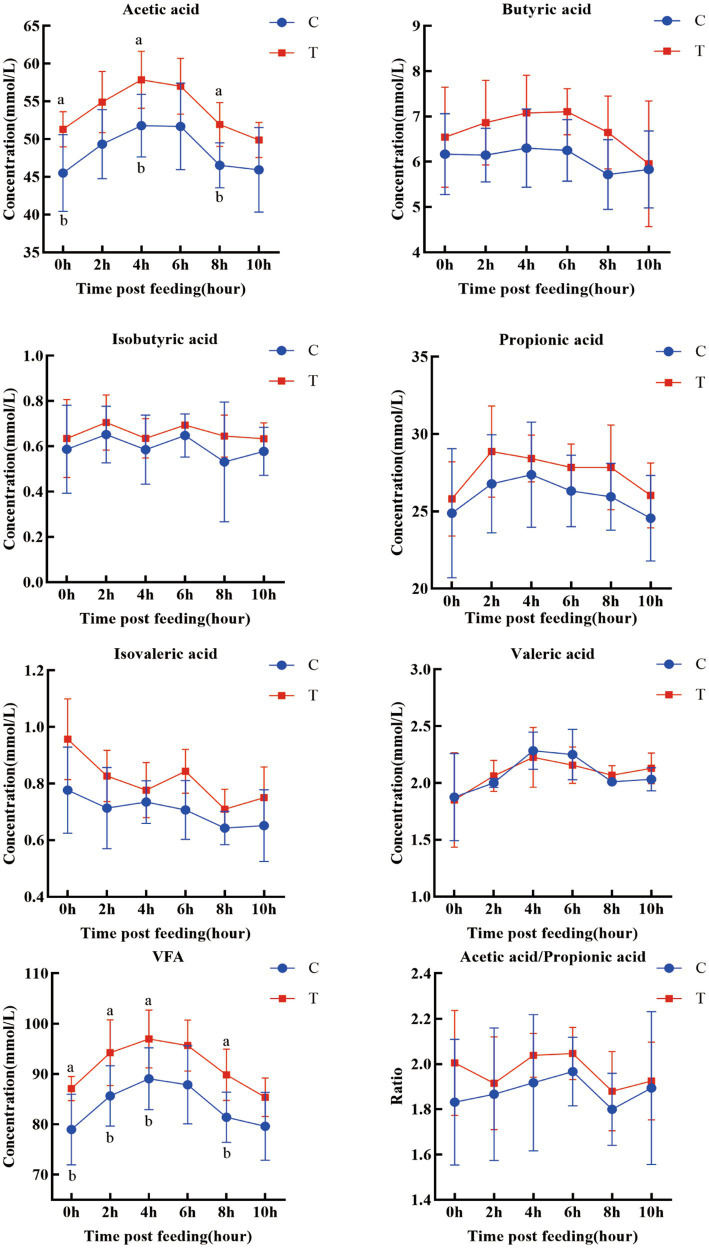
Temporal variations in ruminal VFAs at different time points. a, b means within a figure with different superscripts differ significantly (*p* < 0.05).

The postprandial ruminal VFA concentrations at different time points are shown in Figure 2. The acetate and total VFA concentrations in the trial group were significantly elevated at multiple time points (acetate: 0 h, 4 h, 8 h; total VFA: 0 h, 2 h, 4 h, 8 h) (*p* < 0.05). The acetate-to-propionate ratio was also significantly higher in the trial group (*p* < 0.05). Concentrations of other VFAs (propionate, butyrate, isobutyrate, valerate, isovalerate) showed an increasing trend, but the differences were not significant.

### 3.3 Rumen microbial communities

#### 3.3.1 Alpha diversity of bacterial communities in rumen content

**Figure 3:**
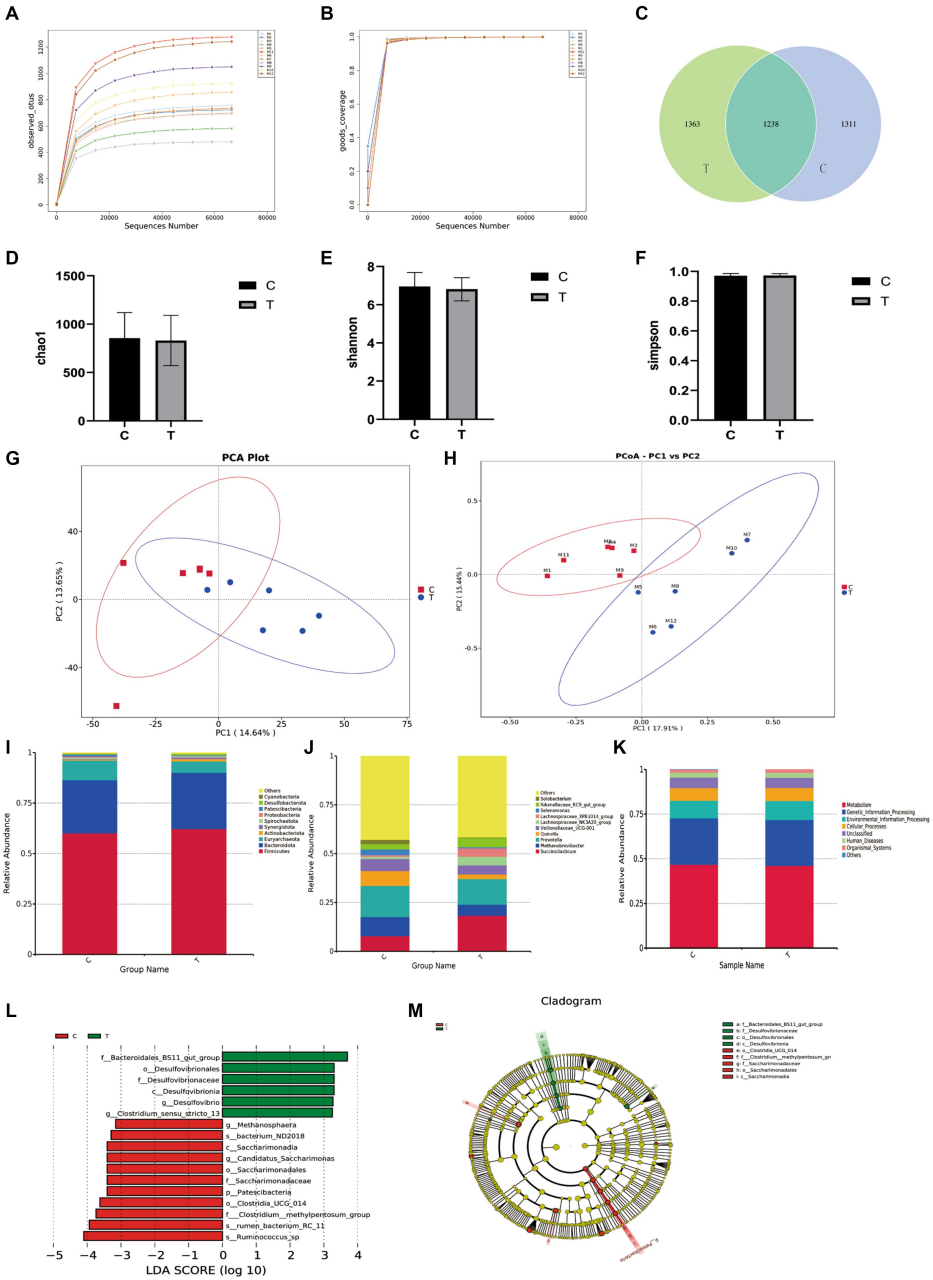
Rumen microbiota results. **(A)** Rarefaction curves of observed species; **(B)** Coverage; **(C)** Venn diagram; **(D)** Chao1 richness estimator; **(E)** Shannon index; **(F)** Simpson index; **(G)** Principal component analysis (PCA); **(H)** Principal coordinate analysis (PCoA); **(I)** Phylum level; **(J)** Genus level; **(K)** Functional prediction analysis; **(L,M)** LEfSe cladogram.

The alpha diversity of ruminal microbiota is illustrated in Figure 3. The sequencing coverage of all samples exceeded 99%, indicating sufficient sequencing depth (Figure 3B). The rarefaction curves plateaued, confirming adequate sequencing volume (Figure 3A). Venn diagram analysis revealed that the number of unique OTUs in the trial group (1,363) was greater than that in the control group (1,311), suggesting a potential increase in species richness (Figure 3C). However, no significant differences were observed in the alpha diversity indices (Chao1, Shannon, Simpson) between groups (*p* > 0.05), indicating that overall microbial diversity and evenness remained unchanged.

#### 3.3.2 Relative abundance of rumen microbiota

At the genus level, detectable taxa in the rumen microbiota included Succiniclasticum, Methanobrevibacter, Prevotella, Quinella, Veillonellaceae_UCG-001, Lachnospiraceae_NK3A20_group, Lachnospiraceae_XPB1014_group, Selenomonas, Rikenellaceae_RC9_gut_group, Solobacterium, with other genera present at low abundance. Compared to the control group, the trial group exhibited: Significantly increased relative abundance of Succiniclasticum (*p* < 0.05). Non-significant increasing trends (*p* > 0.05) in Lachnospiraceae_NK3A20_group, Lachnospiraceae_XPB1014_group, and Rikenellaceae_RC9_gut_group. Nonsignificant decreasing trends (*p* > 0.05) in Methanobrevibacter, Prevotella, Veillonellaceae_UCG-001, Selenomonas, and Solobacterium.

At the phylum level, bacterial phyla detected in the rumen content of sheep included Firmicutes, Bacteroidota, Euryarchaeota, Actinobacteriota, Synergistota, Spirochaetota, Proteobacteria, Patescibacteria, Desulfobacterota, and Cyanobacteria, among others. Compared to the control group, the trial group exhibited a significantly lower relative abundance of Patescibacteria (*p* < 0.05). Changes in the relative abundances of the other phyla were not statistically significant.

At the genus level, detectable genera in the rumen microbiota included *Succiniclasticum*, *Methanobrevibacter*, *Prevotella*, and others. Compared to the control group, the trial group showed a significantly increased relative abundance of *Succiniclasticum* (*p* < 0.05). Non-significant trends of increase or decrease were observed for other genera such as those within Lachnospiraceae.

#### 3.3.3 LEfSe analysis of bacterial communities in rumen content

The LEfSe analysis of bacterial communities in sheep rumen contents revealed significant dominant taxa in the trial group (Figure 3). The LDA scores indicated that the trial group exhibited notable enrichment in the following taxa at specific classification levels: Bacteroidales_BS11_gut_group, Desulfovibrionales, Desulfovibrionaceae, Desulfovibrionia*, Clostridium_sensu_stricto_13*, and *Desulfovibrio*. Additionally, the trial group showed dominance in bacterium_ND2018, Saccharimonadia, Candidatus_Saccharimonas, Saccharimonadales, Saccharimonadaceae, Patescibacteria, Clostridia_UCG_014 (class), Clostridium__methylpentosum_group, RC-11rumen_bacterium_RC_11, and *Ruminococcus_sp*.

Tax4Fun functional prediction indicated that metabolic pathways represented the most abundant functional category, followed by other categories including genetic information processing and environmental information processing. However, no significant differences were observed between the experimental and control groups in these functional categories (*p* > 0.05).

### 3.4 Rumen metabolome

#### 3.4.1 LC–MS-based quality analysis of metabolite samples

**Figure 4:**
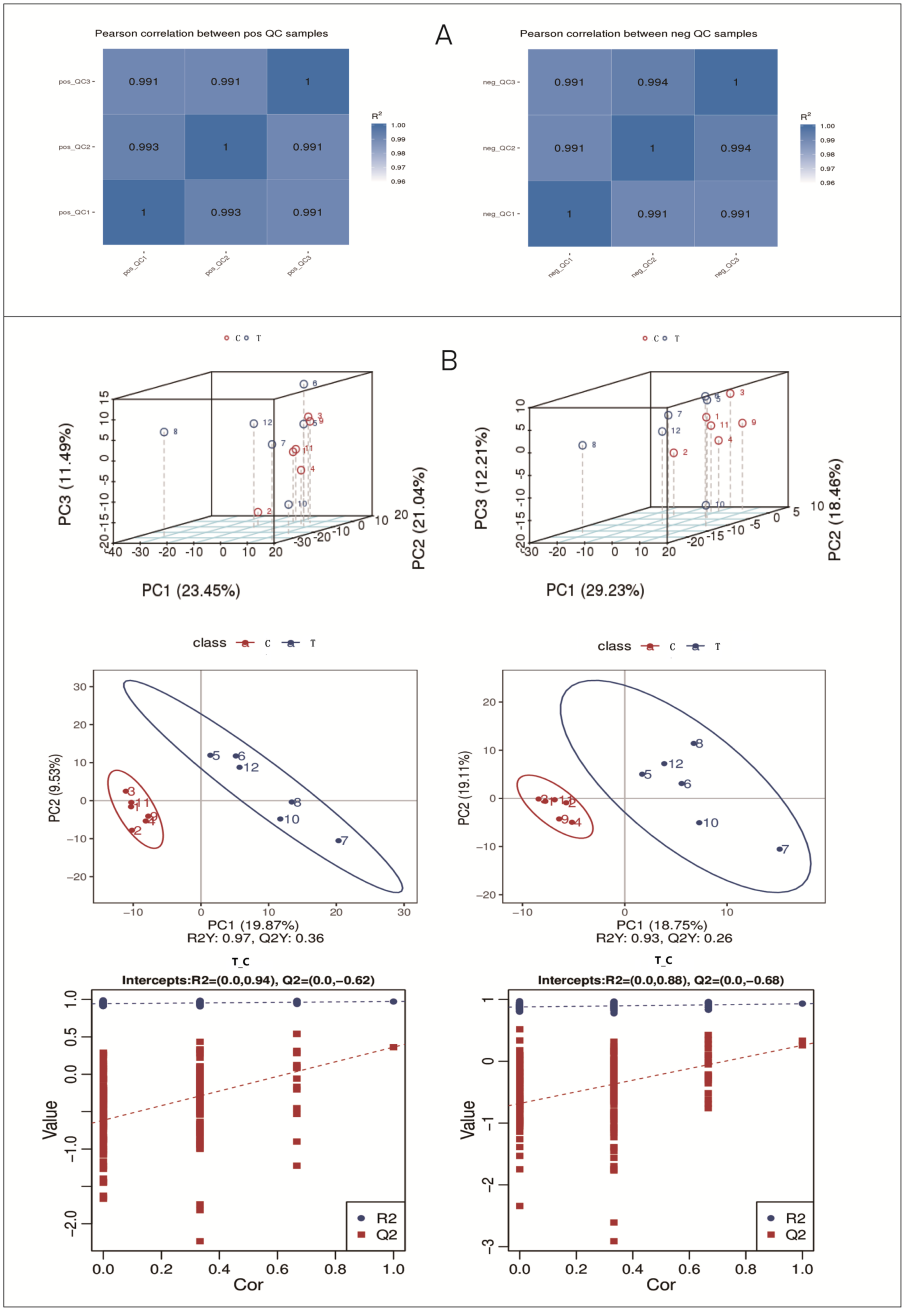
Results of QC sample correlation and PCA analysis. **(A)** Positive ion mode; **(B)** Negative ion mode.

The quality control (QC) chart for the metabolite sample quality analysis is presented in Figure 4. The correlations of QC samples exceeded 0.99 in both positive and negative ion modes. These results indicate high stability of the metabolite detection process and superior data quality, fulfilling the prerequisites for subsequent research data analysis.

#### 3.4.2 Multivariate analysis of rumen metabolome

The PCA results (Figure 4.) of the total samples revealed that in the positive ion mode, the contribution rates of PC1, PC2, and PC3 were 23.45, 21.04, and 11.49%, respectively. In the negative ion mode, the contribution rates of PC1, PC2, and PC3 were 29.23, 18.46, and 12.21%, respectively.

As shown in Figure 4, the PLS-DA model clearly distinguished the metabolomic profiles between the two groups, with high model validity (*R*^2^Y and *Q*^2^ values; intercept of the *Q*^2^ regression line < 0), indicating that the model is reliable and not overfitted.

#### 3.4.3 Composition of metabolites

**Figure 5:**
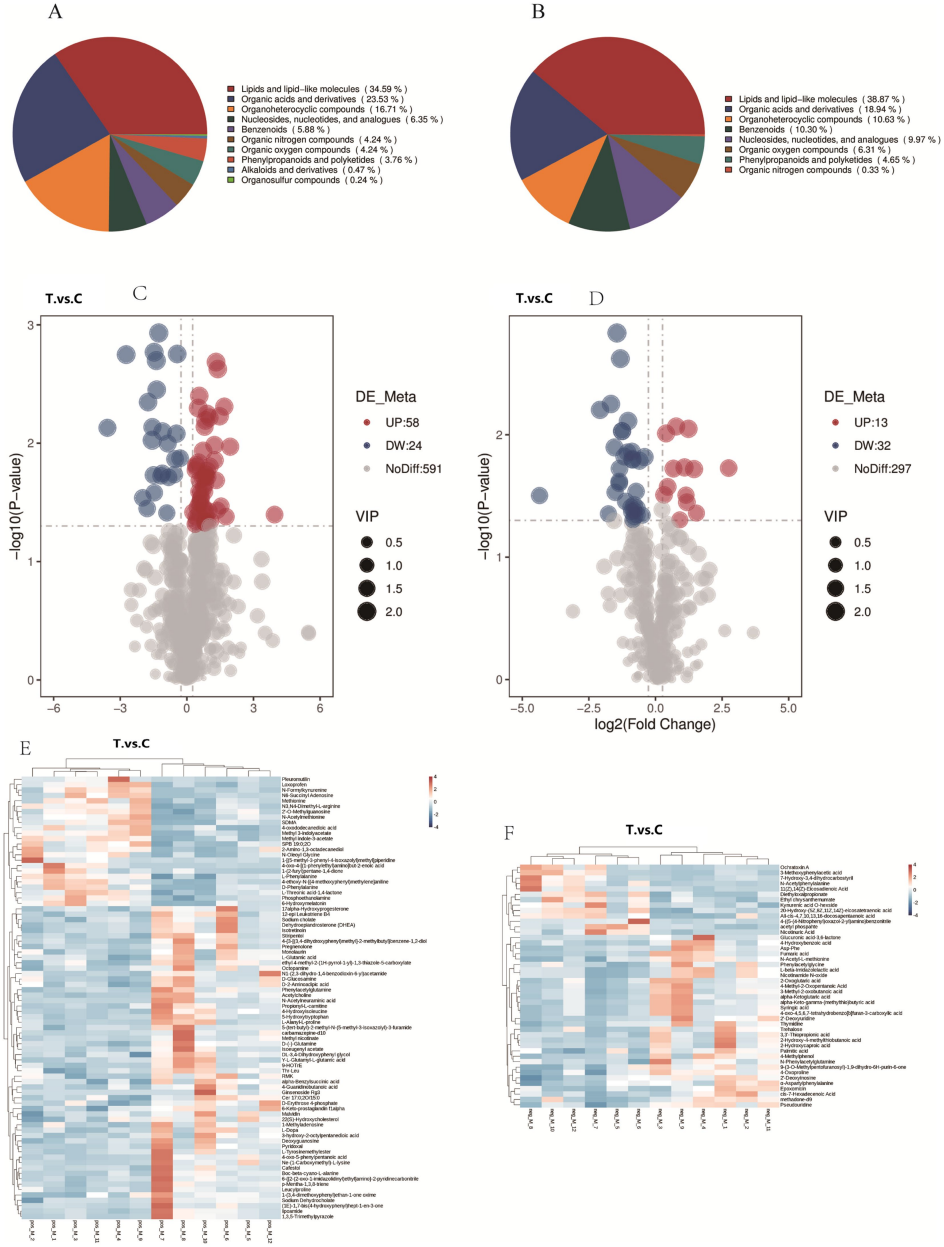
Analysis of rumen metabolites in positive and negative ion modes: proportional distribution **(A,B)**, Metabolite profiles **(C,D)**, and Cluster analysis **(E,F)**.

The classification of rumen metabolites in positive (A) and negative (B) ion modes is shown in Figure 5. In positive ion mode, 10 categories were identified, dominated by lipids and lipid-like molecules (34.59%), organic acids and derivatives (23.53%), and heterocyclic compounds (16.71%). In negative ion mode, 8 categories were identified, primarily lipids and lipid-like molecules (38.87%), organic acids and derivatives (18.94%), and heterocyclic compounds (10.63%).

#### 3.4.4 Screening of differential metabolites

Figure 5 shows the volcano plots of differential metabolites in positive (C) and negative (D) ion modes. Differential metabolites were screened based on the criteria of VIP > 1.0, FC > 1.2 or FC < 1/1.2, and *p*-value < 0.05. A total of 673 metabolites were identified in positive ion mode, among which 82 showed significant differences (58 up-regulated and 23 down-regulated). In negative ion mode, 342 metabolites were identified, with 45 being significantly different (12 up-regulated and 32 down-regulated).

The top 20 upregulated and downregulated differential metabolites were ranked by FC. As shown in Table 3, under positive ion mode: Upregulated metabolites were predominantly saponins, amino acids and derivatives, esters, sugars, heterocyclic compounds, phenolic compounds, and neurotransmitters. Downregulated metabolites included amino acids and derivatives, heterocyclic compounds, hormones and their metabolites, antibiotics, aromatic amines, fatty acids and derivatives, and non-steroidal anti-inflammatory drugs (NSAIDs).

**Table 3:** Metabolites identified in positive ion mode.

Metabolites	Class_I	Class_II	FC	*p*-value	Up/Down
Rg3—Ginsenoside Rg3	Lipids and lipid-like molecules	Prenol lipids	15.48	0.0401	Up
carbamazepine-d10	/	/	3.87	0.0107	Up
N-Acetylneuraminic acid	Organic oxygen compounds	Organooxygen compounds	3.39	0.0417	Up
lipoamide	Organoheterocyclic compounds	Dithiolanes	3.22	0.0049	Up
Isoeugenyl acetate	/	/	2.92	0.0339	Up
1,3,5-Trimethylpyrazole	Organoheterocyclic compounds	Azoles	2.79	0.0059	Up
Phenylacetylglutamine	Organic acids and derivatives	Carboxylic acids and derivatives	2.63	0.0024	Up
(1E)-1,7-bis(4-hydroxyphenyl)hept-1-en-3-one	/	/	2.59	0.0368	Up
1-Methyladenosine	Nucleosides, nucleotides, and analogues	Purine nucleosides	2.50	0.0206	Up
Acetylcholine	Organic nitrogen compounds	Organonitrogen compounds	2.49	0.0021	Up
4-oxo-4-[(1-phenylethyl)amino]but-2-enoic acid	/	/	0.08	0.0074	Down
Pleuromutilin	/	/	0.15	0.0018	Down
1-[(5-methyl-3-phenyl-4-isoxazolyl)methyl]piperidine	/	/	0.25	0.0289	Down
6-Hydroxymelatonin	Organoheterocyclic compounds	Indoles and derivatives	0.29	0.0355	Down
4-ethoxy-N-[(4-methoxyphenyl)methylene]aniline	/	/	0.30	0.0045	Down
4-oxododecanedioic acid	/	/	0.34	0.0094	Down
N6-Succinyl Adenosine	Nucleosides, nucleotides, and analogues	Purine nucleosides	0.34	0.0073	Down
SDMA	Organic acids and derivatives	Carboxylic acids and derivatives	0.35	0.0186	Down
Loxoprofen	Phenylpropanoids and polyketides	Phenylpropanoic acids	0.36	0.0017	Down
1-(2-furyl)pentane-1,4-dione	/	/	0.36	0.0262	Down

From Table 4, under negative ion mode, the upregulated metabolites were predominantly coumarins and derivatives, fatty acids and derivatives, heterocyclic compounds, esters, amino acids and derivatives, and nucleotides and their metabolites. Conversely, the downregulated metabolites primarily included sugars and derivatives, amino acids and derivatives, phenolic compounds, organic acids, and nucleotide derivatives.

**Table 4:** Metabolites identified in negative ion mode.

Metabolites	Class_I	Class_II	FC	*p*-value	Up/Down
Ochratoxin A	Phenylpropanoids and polyketides	Ochratoxins and related substances	6.71	0.0187	Up
7-Hydroxy-3,4-dihydrocarbostyril	Organoheterocyclic compounds	Quinolines and derivatives	2.91	0.0436	Up
11(Z),14(Z)-Eicosadienoic Acid	Lipids and lipid-like molecules	Fatty Acyls	2.73	0.0191	Up
All-cis-4,7,10,13,16-docosapentaenoic acid	Lipids and lipid-like molecules	Fatty Acyls	2.36	0.0089	Up
4-((5-(4-Nitrophenyl)oxazol-2-yl)amino)benzonitrile	Organoheterocyclic compounds	Azoles	2.26	0.0355	Up
Ethyl chrysanthemumate	Lipids and lipid-like molecules	Prenol lipids	2.21	0.0311	Up
N-Acetylphenylalanine	Organic acids and derivatives	Carboxylic acids and derivatives	2.13	0.0185	Up
Nicotinuric Acid	Organic acids and derivatives	Carboxylic acids and derivatives	1.88	0.0495	Up
0-Hydroxy-(5Z,8Z,11Z,14Z)-eicosatetraenoic acid-20	Lipids and lipid-like molecules	Fatty Acyls	1.71	0.0086	Up
acetyl phospahte	/	/	1.60	0.0189	Up
Glucuronic acid-3,6-lactone	Organoheterocyclic compounds	Lactones	0.05	0.0313	Down
2-Hydroxy-4-methylthiobutanoic acid	Lipids and lipid-like molecules	Fatty Acyls	0.24	0.0063	Down
2-Hydroxycaproic acid	Lipids and lipid-like molecules	Fatty Acyls	0.29	0.0442	Down
4-Methylphenol	Benzenoids	Phenols	0.31	0.0056	Down
alpha-Keto-gamma-(methylthio)butyric acid	Lipids and lipid-like molecules	Fatty Acyls	0.34	0.0127	Down
3,3'-Thiopropionic acid	Organic acids and derivatives	Carboxylic acids and derivatives	0.35	0.0295	Down
Fumaric acid	Organic acids and derivatives	Carboxylic acids and derivatives	0.37	0.0015	Down
Nicotinamide N-oxide	/	/	0.39	0.0248	Down
L-beta-Imidazolelactic acid	Organoheterocyclic compounds	Azoles	0.39	0.0241	Down
alpha-Ketoglutaric acid	Organic acids and derivatives	Keto acids and derivatives	0.39	0.0189	Down

#### 3.4.5 Analysis of differential metabolites

**Figure 6:**
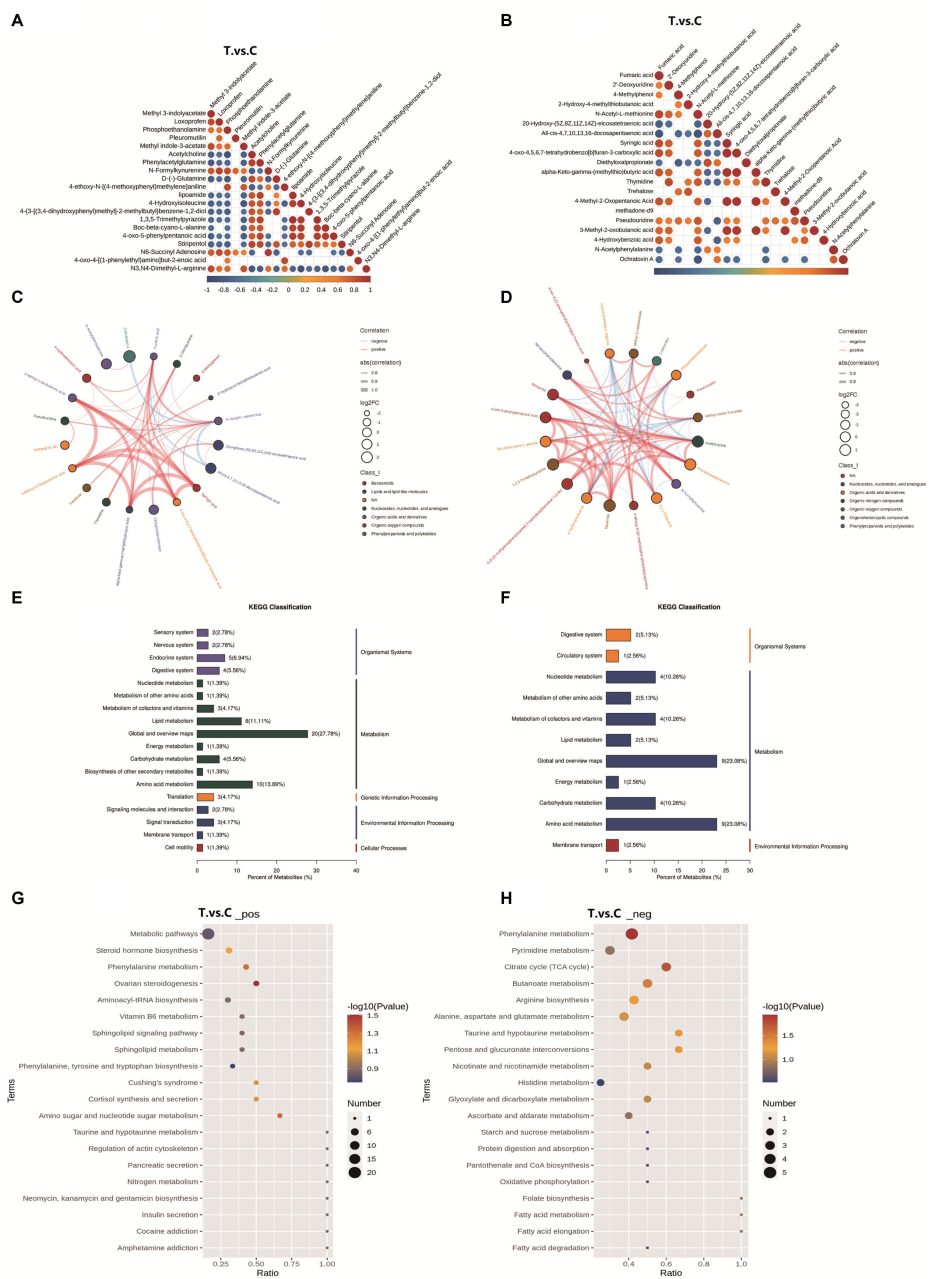
Analysis of differential metabolites: correlation, KEGG classification, and pathway enrichment. **(A,C)** Positive ion mode correlation; **(B,D)** Negative ion mode correlation; The color gradient ranges from red (perfect positive correlation, coefficient = 1) to blue (perfect negative correlation, coefficient = −1), with uncolored regions indicating nonsignificant correlations (*p*-value > 0.05). The top 20 differential metabolites, ranked by ascending *p*-value, are displayed in the chord diagrams. **(E)** KEGG classification in positive mode; **(F)** KEGG classification in negative mode; the horizontal axis represents the percentage of metabolites annotated to a specific KEGG pathway relative to all annotated metabolites. The vertical axis on the right indicates the primary KEGG Pathway categories, while the left side lists the secondary subcategories. **(G)** Pathway enrichment in positive mode; **(H)** Pathway enrichment. The horizontal axis represents the ratio of differential metabolites to total metabolites identified in the corresponding pathway (*x*/*y*). A higher ratio indicates stronger enrichment of differential metabolites in the pathway. The color gradient of the bubbles corresponds to the *p*-value from the hypergeometric test (darker hues denote lower *p*-values, reflecting greater statistical significance). The bubble size correlates with the number of differential metabolites in the pathway (larger bubbles indicate more metabolites).

The cluster analysis of differential metabolites is presented in Figure 6. Color intensity correlates with metabolite abundance, where darker hues indicate higher expression levels and lighter hues represent lower expression levels. The results demonstrated that dietary supplementation with AOC significantly altered rumen microbial metabolites in sheep.

The correlation analysis plots (A/C: positive ion mode; B/D: negative ion mode) and chord diagrams of differential metabolites are shown in Figure 6. In the positive ion mode, metabolites with significant correlations included methyl 3-indolyacetate, methyl indole-3-acetate, loxoprofen, pleuromutilin, phosphoethanolamine, D-(−)-glutamine, and 4-ethoxy-N-[(4-methoxyphenyl)methylene]aniline. In the negative ion mode, significantly correlated metabolites were primarily N-acetyl-L-methionine, ochratoxin A, thymidine, pseudouridine, all-cis-4,7,10,13,16-docosapentaenoic acid, tréhalose, and methadone-d9.

#### 3.4.6 KEGG analysis of differential metabolites

Based on the metabolite classification information provided by the KEGG database, statistical visualization of annotated differential metabolites was performed Figure 6. In positive ion mode, the differential metabolites were widely involved in multiple biological layers, including Organismal Systems (e.g., endocrine and digestive systems), Metabolism (e.g., lipid and amino acid metabolism), Genetic Information Processing, and Environmental Information Processing. Among these, Global and Overview Maps accounted for the highest proportion (27.78%), followed by Amino acid metabolism (13.89%) and Lipid metabolism (11.11%), indicating that these broad metabolic processes were significantly influenced. In negative ion mode, the differential metabolites were more concentrated in the domain of Metabolism, particularly in Amino acid metabolism (23.08%), Nucleotide metabolism (10.26%), and Carbohydrate metabolism (10.26%).

KEGG pathway enrichment analysis was performed to identify the primary biological functions of differential metabolites. Based on the enrichment results, bubble plots of the enriched KEGG pathways were generated Figure 6. In the positive ion mode, differential metabolites were predominantly enriched in metabolic pathways, steroid hormone biosynthesis, phenylalanine metabolism, ovarian steroidogenesis, and aminoacyl-tRNA biosynthesis pathways among the top 20 KEGG pathways. Under negative ion mode, significant enrichment was observed in phenylalanine metabolism, pyrimidine metabolism, citrate cycle (tricarboxylic acid cycle, TCA cycle), butanoate metabolism, arginine biosynthesis, and alanine, aspartate, and glutamate metabolism pathways. These findings highlight critical roles in steroidogenesis, amino acid and nucleotide metabolism, and energy homeostasis, aligning with the observed metabolic shifts in the trial group.

#### 3.4.7 Correlation analysis between key differential metabolites and microbial taxa

**Figure 7:**
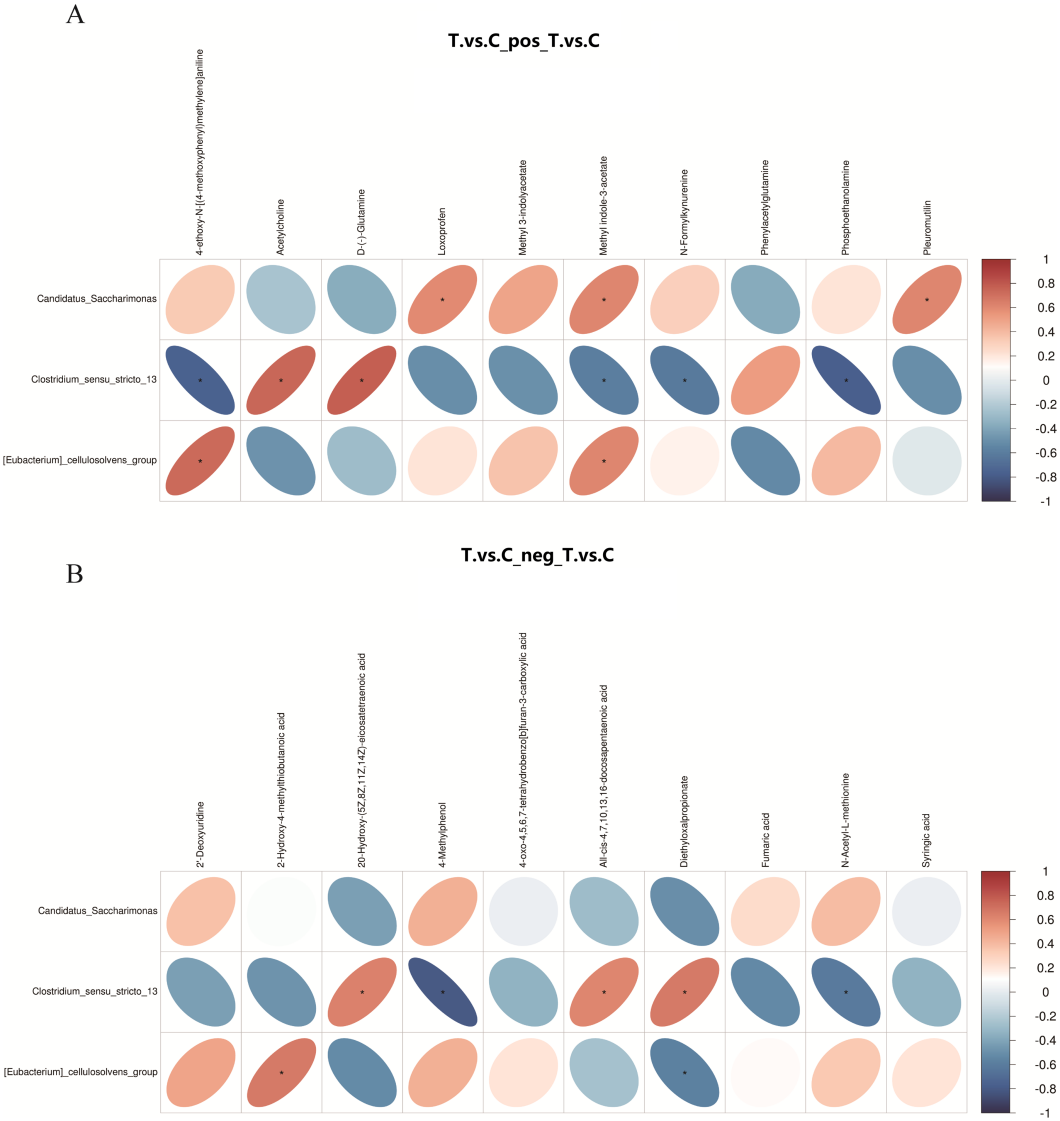
Correlation heatmap between ruminal microbiota and differential metabolites in positive ion **(A)** and negative ion **(B)** modes. The heatmap displays genus-level differential microbiota and significantly altered metabolites, with the color legend indicating Pearson correlation coefficients: red for positive correlations, blue for negative correlations, and asterisks (*) marking statistically significant associations (*p* < 0.05).

Pearson correlation analysis was performed between differential bacterial genera at the genus level and differential metabolites (Figure 7). In positive ion mode (Figure A), *Candidatus Saccharimona* showed a significant positive correlation with anti-inflammatory agents such as loxoprofen. *Clostridium_sensu_stricto_13* was positively correlated with the neurotransmitter acetylcholine, but negatively correlated with metabolites including methyl indole-3-acetate. In negative ion mode, *Clostridium_sensu_stricto_13* demonstrated positive correlations with multiple fatty acids (e.g., eicosatetraenoic acid and docosapentaenoic acid), and a negative correlation with 4-methylphenol.

## 4 Discussion

The results of this study demonstrate that although dietary supplementation with AOC did not significantly affect feed intake in sheep, it significantly increased the average daily gain and reduced the feed-to-gain ratio. These findings are consistent with those reported by Tricarico et al. (13) in beef cattle, further confirming the positive role of *A. oryzae* in improving growth performance in ruminants.

The growth-promoting effect of AOC is closely related to its ability to improve nutrient digestibility. The trial group showed significantly increased neutral detergent fiber (NDF) digestibility, along with upward trends in dry matter, crude protein, and acid detergent fiber (ADF) digestibility. This improvement is primarily attributed to the degradation of fibrous and protein matrices by exogenous enzymes such as cellulases and proteases secreted by AOC (10, 14), a finding that aligns with reports by Hymes-Fecht et al. (6) and Rakhmani et al. (15). Additionally, AOC optimized nitrogen metabolism, as evidenced by reduced fecal and urinary nitrogen excretion and significantly increased nitrogen retention rate, indicating enhanced conversion of dietary nitrogen into microbial protein (16), thereby providing crucial support for the observed improvements in growth performance.

In terms of ruminal fermentation, AOC demonstrates dual benefits of stabilizing the fermentation environment and enhancing energy supply. In modern high-concentrate finishing systems, acidosis seriously compromises animal growth performance (17). In this trial, although the pH values in the trial group showed a tendency to be higher than those in the control group, the difference was not statistically significant. This result is consistent with the findings of Yohe et al. (18) and Chiquette et al. (19), suggesting that supplementation with *A. oryzae* culture may help stabilize ruminal pH under high-concentrate diet conditions. Furthermore, during ruminal fermentation, fibrous feeds are primarily metabolized into VFAs, methane, and carbon dioxide (20). The results of this trial showed that the ruminal acetate concentration and total VFA content in the trial group were significantly higher than those in the control group, with other VFAs such as propionate also exhibiting an increasing trend. These findings are consistent with reports by Zhang et al. (21) and Cantet et al. (22), indicating that supplementation with *A. oryzae* culture significantly enhances ruminal fermentation efficiency. Combined with the improved NDF digestibility, it can be inferred that *A. oryzae* promotes VFA production by enhancing fiber degradation and microbial fermentation activity. The resulting increase in VFA generation provides more metabolizable energy to the host (23, 24), which aligns well with the observed improvement in average daily gain.

Analysis of the ruminal microbial community using high-throughput sequencing revealed that the trial group exhibited a higher OTU count than the control group following supplementation with *A. oryzae* culture. However, no significant differences were observed in the Chao1, Shannon, or Simpson indices between the groups, indicating that AOC supplementation did not significantly alter microbial community diversity.

Previous studies have demonstrated that that supplementing sheep diets with 2 g/d of *A. oryzae* fermented extract significantly increased the total bacterial number and the number of cellulolytic bacteria in the rumen fluid by 34 and 90%, respectively Newbold et al. (25). Further supporting this, Sun et al. (26) confirmed that adding AOC to the diet of cannulated dairy cows significantly increased the abundances of *Ruminococcus albus* and *Ruminococcus flavefaciens*. The results of this trial showed an increasing trend in the abundances of the phyla Firmicutes and Bacteroidetes in the trial group compared to the control group, which is consistent with the findings of Zhang Duihong et al. (21), indicating that AOC promotes these two dominant phyla in the sheep rumen (48). At the genus level, the trial group exhibited increasing trends in *Succiniclasticum* abundance compared to the control group. *Succiniclasticum*, a key propionate-producing genus, participates in hemicellulose degradation and converts succinate to propionate (27), aligning with the observed rise in propionate proportion within ruminal volatile fatty acids. Additionally, *Lachnospiraceae_NK3A20_group* and *Lachnospiraceae_XPB1014_group* were enriched in the trial group, consistent with Piao et al. (28), who reported similar trends in sheep supplemented with *A. oryzae* culture. These taxa are implicated in lignocellulose breakdown (50), suggesting that *A. oryzae* enhances fibrolytic microbial populations (29, 47).

Notably, the trial group showed significantly lower abundance of Patescibacteria—a phylum often associated with dysbiosis (30). A reduced trend in Proteobacteria, which is linked to gut microbial instability (31). These shifts suggest that *A. oryzae* supplementation enhances rumen microbial homeostasis by enriching beneficial taxa (e.g., fiber degraders) while suppressing potential pathobionts. The trial group also showed a rising trend in *Rikenellaceae_RC9_gut_group,* a dominant gut microbiota phylotype associated with mucin metabolism (32), negative correlation with obesity (33), carbohydrate digestion (34), and anti-inflammatory effects via suppression of pro-inflammatory cytokines (35). This group enhances mucosal barrier integrity by elevating butyrate levels, a critical regulator of intestinal health (36), indicating that *A. oryzae* supplementation may improve rumen epithelial function through microbiota modulation. The synergistic effect between the enrichment of beneficial microbes (such as *Rikenellaceae_RC9_gut_group*) and the upregulation of anti-inflammatory metabolites (such as EPA) aligns with the “microbiota-immunity-barrier” axis regulation mechanism proposed by Su et al. (37). Their study indicates that functional additives, by modulating the gut microbiota, not only promote the production of beneficial metabolites like short-chain fatty acids but also directly enhance intestinal epithelial barrier integrity and regulate the host immune system. This provides robust theoretical support and a mechanistic explanation for the hypothesis that AOC improves rumen health through “microbiota-metabolite” interactions. LEfSe analysis further identified *Bacteroidales_BS11_gut_group* and *Desulfovibrio* as discriminant taxa in the trial group. *Bacteroidales_BS11_gut_group* is linked to sulfur metabolism and anti-inflammatory activity, potentially explaining the upregulation of antioxidant metabolites like lipoic acid in metabolomic profiles. *Desulfovibrio*, a sulfate-reducing genus, may contribute to sulfur cycling, synergizing with enhanced antioxidant pathways to bolster rumen health.

Non-targeted metabolomics elucidated the mechanism of AOC action at the molecular level. Differential metabolites were primarily enriched in pathways such as phenylalanine metabolism, the tricarboxylic acid (TCA) cycle, and lipid metabolism. The activation of these pathways signifies enhanced host energy metabolism (38) and improved nitrogen utilization efficiency (39, 40), which aligns with the aforementioned findings of increased VFA production and nitrogen retention. Metabolites of *Aspergillus oryzae* (such as polysaccharides and polyphenols) can directly scavenge free radicals or upregulate the expression of antioxidant enzymes like SOD and CAT (10). Metabolomic data from this trial revealed significant upregulation of antioxidants including ginsenoside Rg3 and lipoic acid (41, 42), as well as anti-inflammatory metabolites such as eicosapentaenoic acid (EPA) and nicotinuric acid (43, 44). Concurrently, the downregulation of the anti-inflammatory drug loxoprofen may indicate alleviated systemic oxidative stress (45). This finding aligns with the research conclusions of Chen et al. (46) on natural plant-derived additives, whose study confirmed that such additives can effectively elevate the levels of antioxidant metabolites in the body, thereby enhancing the animal’s resistance to physiological stress. Furthermore, computational approaches such as microbiome-disease association analysis and network modeling enable the prediction of key functional microbes and core metabolic pathways from correlative data (11). In the present trial, the observed increase in beneficial microbes and reduction in potential pathogens within the microbiome suggest that AOC may synergistically improve the rumen’s antioxidant and anti-inflammatory status through “microbe-metabolite” interactions, ultimately promoting healthier growth in sheep.

This study observed the growth-promoting effects of AOC in sheep through production performance indicators, and further revealed its potential mechanisms through ruminal microbiota and metabolomic analyses. However, several limitations should be considered: the relatively small sample size (only 6 sheep per group) may limit the statistical power of the findings; the 30-day trial duration prevented assessment of long-term effects; methane emissions were not measured, restricting comprehensive evaluation of environmental impact; and the lack of functional validation data made it difficult to establish causal relationships between microbial functions and metabolic phenotypes. Future studies should expand the sample size, extend the trial period, and integrate multi-omics data with functional validation to further elucidate the mechanisms of AOC.

## 5 Conclusion

Dietary supplementation with 1% AOC improved rumen fermentation efficiency in sheep through synergistic modulation of multiple metabolic pathways, including lipid metabolism, amino acid metabolism, and the TCA cycle, thereby enhancing energy utilization. The increased abundance of fiber-degrading taxa (e.g., *Lachnospiraceae groups*) promoted fibrous diet degradation, contributing to enhanced production performance. Furthermore, upregulation of antioxidant metabolites (e.g., ginsenoside Rg3, lipoic acid) and downregulation of inflammatory mediators (e.g., loxoprofen) indicated improved rumen health and stress resilience. These findings demonstrate that AOC optimizes rumen microbiota functionality and metabolic homeostasis, offering a sustainable strategy to enhance sheep productivity and welfare.

## Data Availability

The data presented in this study are deposited in the NCBI BioProject repository, accession number PRJNA1348942, and can be accessed at: http://www.ncbi.nlm.nih.gov/bioproject/1348942.
