# Supplementary material for: Metabolomic and microbial diversity perspectives on Aspergillus oryzae culture-induced modifications in ovine feed utilization and rumen ecosystem
Source: Front Vet Sci. 2025 Nov 14;12:1658361. doi: 10.3389/fvets.2025.1658361 (PMC12662177; doi:10.3389/fvets.2025.1658361)
Supplement: Supplementary file 1 [file Presentation_1.pdf]

Appendix 1.

The moisture content in the diet and feces was determined according to the method specified in GB/T 6435-2014. The specific procedure was as follows: A clean weighing bottle was placed in a drying oven at 103 ± 2 ℃ with its lid removed and placed beside the bottle. After drying for 30 minutes, the lid was placed back on, and the bottle was transferred to a desiccator to cool down to room temperature. Its mass was weighed and recorded as m_1 with an accuracy of 1 mg. Approximately 5 g of sample (m_2) was weighed into the weighing bottle with an accuracy of 1 mg. The weighing bottle (without the lid, which was placed beside it) was then placed in the drying oven at 103 ± 2 ℃. Once the temperature stabilized at 103 ± 2 ℃, the sample was dried for 4 hours. Afterwards, the lid was placed back on, and the bottle was transferred to a desiccator to cool to room temperature. Its mass was weighed and recorded as m_3 with an accuracy of 1 mg. Subsequently, the bottle was dried again in the oven at 103 ± 2 ℃ for 30 ± 1 minutes, removed, cooled in the desiccator to room temperature, and weighed again with an accuracy of 1 mg. If the change in mass between these two successive weighings was less than or equal to 0.1% of the sample mass, the first mass value (m_3) was used for calculation. The moisture content was calculated using the following formula: Moisture (%) = $[m_2 - (m_3 - m_1)] / m_2 \times 100\%$.

The CP content in the diet, feces, and urine samples was determined according to the method specified in GB/T 6432-2018. The detailed procedure was as follows: Digestion was carried out in digestion tubes, with two parallel replicates per sample. A sample weighing between 0.5 g and 2 g was placed into a digestion tube. Then, 2 Kjeldahl catalyst tablets and 12 mL of sulfuric acid were added. The mixture was digested at 420 ℃ on a digestion furnace for 1 hour. After digestion, the tube was removed and allowed to cool to room temperature. Subsequently, 20 mL of water was added, and the solution was transferred into a 100 mL volumetric flask. After cooling, the volume was made up to the mark with water and mixed thoroughly to obtain the sample digest solution. The condenser tip of a semi-micro distillation unit was immersed into a

conical flask containing 20 mL of boric acid absorption solution (prepared by dissolving 20 g of boric acid in water and diluting to 1000 mL) and 2 drops of mixed indicator (a 1:1 volumetric mixture of methyl red ethanol solution and bromocresol green ethanol solution). Several drops of methyl red indicator (prepared by dissolving 0.1 g of methyl red in ethanol and diluting to 100 mL) and a few drops of sulfuric acid were added to the water in the steam generator. The solution in the steam generator was maintained as orange-red during distillation; additional sulfuric acid was supplemented if necessary. A volume of 10 mL to 20 mL of the sample digest solution was accurately pipetted into the reaction chamber of the distillation unit. The inlet was rinsed with a small amount of water and sealed with the glass stopper. Then, 10 mL of sodium hydroxide solution (prepared by dissolving 40 g of sodium hydroxide in water, cooling to room temperature, and diluting to 100 mL with water) was added by carefully lifting the glass stopper to allow the solution to flow into the reaction chamber. The stopper was resealed securely, and water was added at the inlet to ensure an airtight seal. Distillation was conducted for 4 minutes. The conical flask was then lowered so that the condenser tip was above the surface of the absorption solution, and distillation continued for another 1 minute until the pH of the distillate became neutral. The condenser tip was rinsed with water, collecting all washings into the conical flask, before stopping the distillation. The resulting absorption solution was immediately titrated with 0.1 mol/L hydrochloric acid (HCl) standard titration solution. The endpoint was indicated by a color change from blue-green to dull red.

The crude protein content was calculated using the following formula: $CP (\%) = [((V_2 - V_1) \times C \times (14 / 1000) \times 6.25) / (m \times (V_3 / V_4))] \times 100$. V_1 is the volume (mL) of HCl standard titration solution consumed in the blank titration, V_2 is the volume (mL) of HCl standard titration solution consumed in the sample titration, V_3 is the volume (mL) of the digest solution used for distillation, V_4 is the total volume (mL) of the digest solution, C is the concentration (mol/L) of the HCl standard titration solution, and m is the mass (g) of the sample.

The neutral detergent fiber (NDF) and acid detergent fiber (ADF) contents in the diet and feces were determined using a fully automated fiber analyzer (ANKOM A2000i).

Approximately 0.5 g of sample was weighed into filter bags, which were numbered with a solvent-resistant marker and their weights recorded before being heat-sealed. The sealed bags were placed into the instrument, which automatically dispensed neutral detergent solution. During the filling of the tank, 4 mL of thermo-stable α-amylase was added, and the tank was tightly closed. After approximately 1.5 hours, when the instrument indicated the completion of washing, the bags were removed, drained thoroughly, immersed in acetone for 3–5 minutes, squeezed to remove excess liquid, and placed in a fume hood until the acetone had completely evaporated. They were then dried at 105 °C for 3 hours. The dried bags were transferred to a desiccator to cool and subsequently weighed. The composition of the solutions used followed the Chinese National Standard GB/T 20806—2006. The NDF content was calculated using the following formula: NDF (%) = $[m_2 - (m_1 \times C_1)] \times 100 / m$. m is the mass (g) of the sample, m_1 is the initial mass (g) of the empty bag, m_2 is the mass (g) of the residue plus the bag after extraction, C_1 is the blank bag correction factor (calculated as the mass after drying divided by the original mass).

After NDF determination, the bags containing the residue were placed directly on the sample holder for ADF analysis. Acid detergent solution was added, and the instrument was set to acid washing mode. After approximately 1 hour, when the instrument indicated the end of washing, the bags were removed, drained, immersed in acetone for 3–5 minutes, squeezed, and allowed to dry in a fume hood until all acetone had evaporated. They were then dried at 105 °C for 3 hours, cooled in a desiccator, and weighed. The composition of the solutions used for ADF determination adhered to the Chinese Agricultural Industry Standard NY/T 1459—2007. The ADF content was calculated as follows: ADF (%) = $[(m_3 - m_4) / m] \times 100$ m_3 is the mass (g) of the bag plus ADF residue after washing, m_4 is the mass (g) of the bag, m is the mass (g) of the sample.

Appendix 2

Bacterial diversity was assessed using primers targeting the V4 region of the 16S rRNA gene (515F forward primer and 806R reverse primer). All PCR reactions were carried out with 15 μL of Phusion® High- Fidelity PCR Master Mix (New England Biolabs); 0.2 μM of forward and reverse primers, and about 10 ng template DNA. Thermal cycling consisted of initial denaturation at 98°C for 1 min, followed by 30 cycles of denaturation at 98°C for 10 s, annealing at 50°C for 30 s, and elongation at 72°C for 30 s and 72°C for 5 min. The PCR products were purified using magnetic bead purification. Samples were mixed in equidensity ratios based on the concentration of PCR products. After thorough mixing, the PCR products were detected and target bands were recovered. Sequencing libraries were generated and indexes were added. The library was checked with Qubit and real-time PCR for quantification and bioanalyzer for size distribution detection. Quantified libraries were pooled and sequenced on Illumina platforms, according to effective library concentration and data amount required. Paired- end reads was assigned to samples based on their unique barcode and truncated by cutting off the barcode and primer sequence. Paired-end reads were merged using FLASH (V1.2. 1 1, http://ccb.jhu.edu/software/FLASH/), a very fast and accurate analysistool, which was designed to merge paired- end reads when at least some of the reads overlap theread generated from the opposite end of the same DNA fragment, and the splicing sequences were called raw tags. Quality filtering on the raw tags were performed using the fastp (Version 0 .23 . 1) software to obtain high-quality Clean Tags. The tags were compared with the reference database (Silva database (16S/18S),

https://www.arb-silva.de/; Unite Database (ITS) , https://unite.ut.ee/) to detect chimera sequences, And the effective tags were obtained by removing the chimera sequences with the vsearch package (V2.16.0, https://github.com/torognes/vsearch). For the Effective Tags obtained previously, denoise was performed with DADA2 or deblur module in the QIIME2 software (Version QIIME2-202202) to obtain initial ASVs (Amplicon Sequence Variants) (default: DADA2). Species annotation was performed using QIIME2 software. For 16S/18S, the annotation database is Silva Database, while for ITS, it is Unite Database. For unregular region, Unregular region: Micro_NT by default . In order to analyze the diversity, richness and uniformity of the communities in the sample, alpha diversity was calculated from 7 indices in QIIME2, including Observed_otus, Chao1, Shannon, Simpson, Dominance, Good's coverage and Pielou_e.

Appendix 3.

The samples (100 μL) were placed in the EP tubes and resuspended with prechilled 80% methanol by well vortex. Then the samples were incubated on ice for 5 min and centrifuged at 15,000 g, 4°C for 20 min. Some of supernatant was diluted to final concentration containing 53% methanol by LC-MS grade water. The samples were system analysis. Cell or bacteria sample subsequently transferred to a fresh Eppendorf tube and then were centrifuged at 15000 g, 4°C for 20 min. Finally, the supernatant was injected into the LC-MS/MS. UHPLC-MS/MS analyses were performed using a Vanquish UHPLC system (ThermoFisher, Germany) coupled with an Orbitrap Q ExactiveTM HF mass spectrometer or Orbitrap Q ExactiveTMHF-X mass spectrometer (Thermo Fisher, Germany) in Novogene Co., Ltd. (Beijing, China). Samples were injected onto a Hypersil Goldcolumn (100×2.1 mm, 1.9μm) using a 12-min linear gradient at a flow rate of 0.2 mL/min. The eluents for the positive and negative polarity modes were eluent A (0.1% FA in Water) and eluent B (Methanol). The solvent gradient was set as follows: 2% B, 1.5 min; 2-85% B, 3 min; 85-100% B, 10 min; 100-2% B, 10.1 min; 2% B, 12 min. Q ExactiveTM HF mass spectrometer was operated in positive/negative polarity mode with spray voltage of 3.5 kV, capillary temperature of 320°C, sheath gas flow rate of 35 psi and aux gas flow rate of 10 L/min, S-lens RF level of 60, Aux gas heater temperature of 350°C.

The raw data files generated by UHPLC-MS/MS were processed using the Compound Discoverer 3.3 (CD3.3, ThermoFisher) to perform peak alignment, peak picking, and quantitation for each metabolite. The main parameterswere set as follows: peak area was corrected with the first QC, actual mass tolerance, 5ppm; signal intensity tolerance, 30%; and minimum intensity, et al. After that, peak intensities were normalized to the total spectral intensity. The normalized data was used to predict the molecular formula based on additive ions, molecular ion peaks and fragment ions. And then peaks were matched with the mzCloud (https://www.mzcloud.org/)， mzVault and MassList

database to obtain the accurate qualitative and relative quantitative results. Statistical analyses were performed using the statistical software R (R version R-3.4.3), Python (Python 2.7.6 version) and CentOS (CentOS release 6.6), When data were not normally distributed, standardize according to the formula: sample raw quantitation value / (The sum of sample metabolite quantitation value / The sum of QC1 sample metabolite quantitation value) to obtain relative peak areas; And compounds whose CVs of relative peak areas in QC samples were greater than 30% were removed, and finally the metabolites' identification and relative quantification results were obtained.

These metabolites were annotated using the KEGG database (https://www.genome.jp/kegg/pathway.html) , HMDB database (https://hmdb.ca/metabolites) and LIPIDMaps database (http://www.lipidmaps.org/). Principal components analysis (PCA) and Partial least squares discriminant analysis (PLS-DA) were performed at metaX. We applied univariate analysis (t-test) to calculate the statistical significance (P-value).The metabolites with VIP > 1 and P-value< 0.05 and fold change≥2 or FC≤0.5 were considered to be differential metabolites. Volcano plots were used to filter metabolites of interest which based on $\log_2$ (FoldChange) and $-\log_{10}$(p-value) of metabolites by ggplot2 in R language. For clustering heat maps, the data were normalized using z-scores of the intensity areas of differential metabolites and were ploted by Pheatmap package in R language. The correlation between differential metabolites were analyzed by cor () in R language (method=pearson). Statistically significant of correlation between differential metabolites were calculated by cor.mtest() in R language. P-value < 0.05 was considered as statistically significant and correlation plots were ploted by corrplot package in R language.The functions of these metabolites and metabolic pathways were studied using the KEGG database.The metabolic pathways enrichment of differential metabolites was performed, when ratio were satisfied by x/n > y/N, metabolic pathway were considered asenrichment, when

P-value of metabolic pathway < 0.05, metabolic pathway were considered as statistically significant enrichment.

Perform correlation analysis and generate a heatmap based on Pearson correlation coefficients between significantly differential bacterial genera (identified from 16S rDNA or metagenomic analysis) and significantly differential metabolites (identified from metabolomic analysis). This aims to measure the association between species diversity and metabolites in environmental samples. The correlation coefficient ranges from -1 to 1. A coefficient less than 0 indicates a negative correlation; a coefficient greater than 0 indicates a positive correlation; and a coefficient equal to 0 indicates no correlation. The formula for the Pearson correlation coefficient (r) is: $r = cov(X, Y) / (\sigma X * \sigma Y)$, where cov(X, Y) represents the covariance of X and Y, and σX and σY represent the standard deviations of X and Y. The correlation coefficient ranges from -1 to 1. When $r > 0$, it indicates a positive correlation; when $r < 0$, it indicates a negative correlation; and when $r = 0$, it indicates no correlation.
